# Modulating the Adhesion of Haematopoietic Stem Cells with Chemokines to Enhance Their Recruitment to the Ischaemically Injured Murine Kidney

**DOI:** 10.1371/journal.pone.0066489

**Published:** 2013-06-19

**Authors:** Rebecca L. White, Gerard Nash, Dean P. J. Kavanagh, Caroline O. S. Savage, Neena Kalia

**Affiliations:** 1 School of Clinical and Experimental Medicine, College of Medical and Dental Sciences, University of Birmingham, Birmingham, United Kingdom; 2 School of Immunity and Infection, College of Medical and Dental Sciences, University of Birmingham, Birmingham, United Kingdom; UCL Institute of Child Health, United Kingdom

## Abstract

**Introduction:**

Renal disease affects over 500 million people worldwide and is set to increase as treatment options are predominately supportive. Evidence suggests that exogenous haematopoietic stem cells (HSCs) can be of benefit but due to the rarity and poor homing of these cells, benefits are either minor or transitory. Mechanisms governing HSC recruitment to injured renal microcirculation are poorly understood; therefore this study determined (i) the adhesion molecules responsible for HSC recruitment to the injured kidney, (ii) if cytokine HSC pre-treatment can enhance their homing and (iii) the molecular mechanisms accountable for any enhancement.

**Methods:**

Adherent and free-flowing HSCs were determined in an intravital murine model of renal ischaemia-reperfusion injury. Some HSCs and animals were pre-treated prior to HSC infusion with function blocking antibodies, hyaluronidase or cytokines. Changes in surface expression and clustering of HSC adhesion molecules were determined using flow cytometry and confocal microscopy. HSC adhesion to endothelial counter-ligands (VCAM-1, hyaluronan) was determined using static adhesion assays *in vitro*.

**Results:**

CD49d, CD44, VCAM-1 and hyaluronan governed HSC adhesion to the IR-injured kidney. Both KC and SDF-1α pre-treatment strategies significantly increased HSC adhesion within injured kidney, whilst SDF-1α also increased numbers continuing to circulate. SDF-1α and KC did not increase CD49d or CD44 expression but increased HSC adhesion to VCAM-1 and hyaluronan respectively. SDF-1α increased CD49d surface clustering, as well as HSC deformability.

**Conclusion:**

Increasing HSC adhesive capacity for its endothelial counter-ligands, potentially through surface clustering, may explain their enhanced renal retention *in vivo*. Furthermore, increasing HSC deformability through SDF-1α treatment could explain the prolonged systemic circulation; the HSC can therefore continue to survey the damaged tissue instead of becoming entrapped within non-injured sites. Therefore manipulating these mechanisms of HSC recruitment outlined may improve the clinical outcome of cellular therapies for kidney disease.

## Introduction

Acute renal failure (ARF) affects 5% of all hospitalised patients and is one of the leading worldwide causes of morbidity and mortality [1]. A common cause of ARF is ischaemia-reperfusion (IR) injury, which causes renal tubular death, glomerular injury and inflammation [2], [3], [4]. Evidence suggests ARF predisposes a person to accelerated chronic renal failure due to persistent interstitial fibrosis [5] and because of very few highly effective therapies for ARF, the incidence of chronic renal failure is set to increase causing a huge financial strain on society. Although the kidney has a remarkable regenerative capacity [6], this becomes overwhelmed during sustained periods of renal injury. Recent evidence suggests that exogenous bone marrow (BM)-derived haematopoietic stem (HSC) and progenitor (HPC) cells can confer structural and functional benefit following ARF, most likely due to paracrine mechanisms such as secretion of growth factors and inhibition of apoptosis [7], [8], [9]. Other BM-derived cells, such as mesenchymal stem cells (MSC) and endothelial progenitor cells (EPC) have also shown to reduce fibrosis in more chronic renal disease models and clinical trials using BM-derived cells are currently ongoing [10], [11], [12], [13]. This shows that exogenously injected stem cells are of benefit to both acutely and chronically injured mice.

Despite emerging clinical evidence that these stem cells (SCs) can improve a variety of inflammatory disorders, benefits are either minor or transitory [14], [15]. This has been partially explained by low numbers of HSCs actually adhering within the local microcirculation of injured organs after injection [16]. Therefore, when delivered by the preferred systemic route, poor homing and a subsequent low efficiency of tissue engraftment occurs; processes that are essential for SCs to mediate repair [17]. For example, within infarcted heart, progenitor cell retention is less than 5% [18]. Poor homing, combined with the fact that HSCs are rare cells, (<0.01% of BM), has likely limited their clinical utility and success. If SC therapy is to be realised for renal diseases, a better understanding of the adhesive mechanisms underlying their recruitment to the injured kidney is essential. This may enable development of strategies that can enhance this phenomenon and potentially lead to more rapid, efficient and longer lasting tissue repair.

Our knowledge of the adhesive mechanisms mediating recruitment of transplanted HSCs to injured kidney is limited. HSCs possess a similar repertoire of surface adhesion molecules to leukocytes, expressing β_1_- and β_2_-based integrin heterodimers which bind to their endothelial counter-receptors, VCAM-1 and ICAM-1 respectively [19]. Recent work from our group has demonstrated a critical role for the CD49d (α_4_ subunit of α_4_β_1_ integrin)/VCAM-1 pathway in mediating HSC recruitment to injured murine liver [20]. Similar interactions also mediate HSC recruitment to the BM, implicating an important role for this integrin in HSC homing [21]. However, it is not known whether CD49d is universally responsible for retaining HSCs in all injured vascular beds, including the kidney. The non-integrin CD44 has been the subject of a number of studies but its role in stem cell trafficking remains controversial [22], [23], [24], [25]. The main ligand for CD44 is hyaluronic acid (HA), which is expressed in the extracellular matrix of most tissue-beds [26] and is highly expressed in the kidney after IR injury [27]. Therefore, this study initially determined the molecular adhesive mechanisms governing HSC recruitment to IR injured murine renal microcirculation *in vivo*. Specifically, the roles of the integrin sub-units CD18 (β_2_) and CD49d (α4), and the non-integrin CD44 were determined.

Since SC adhesion molecules play an important part in mediating SC-endothelial interactions, modulating their expression and/or binding ability for endothelial counter-ligands might be an important approach to improve renal SC homing and thus potentially enhance the effectiveness of SC therapy. The injured kidney releases an inflammatory milieu that provides an activated environment which could enhance stem cell adhesion to the injured endothelium [13]. The chemical mediator releasate includes chemokines and reactive oxygen species, and these can activate adhesion molecules on trafficking HSCs, in a similar manner to leukocytes, and subsequently initiate their adhesion to microvessels [28]. We recently demonstrated that HSC adhesion within IR injured mouse intestinal microcirculation could be increased by pre-treating them with the reactive oxygen species hydrogen peroxide [29]. This suggests unmanipulated HSC homing is not maximal and thus enhancement of HSC recruitment may be possible. Stromal cell-derived factor-1α (SDF-1α; CXCL12), released following renal injury, is a potent chemokine required for SC homing to BM and non-medullary tissues post-injury [30], [31]. Although keratinocyte-derived chemokine (KC; murine functional homologue of human IL-8) is also released after acute renal injury [32], the exact role of this cytokine in HSC homing to injured sites is unknown. Therefore, the role of these inflammatory chemokines in promoting HSC homing to healthy kidney was investigated. Thereafter, studies were conducted to determine whether, and how, pre-treating HSCs with SDF-1α or KC prior to their infusion could enhance their renal homing efficiency to IR injured kidney.

## Results

### Adherent and free flowing HSC numbers are increased in IR injured kidney

**Figure 1:**
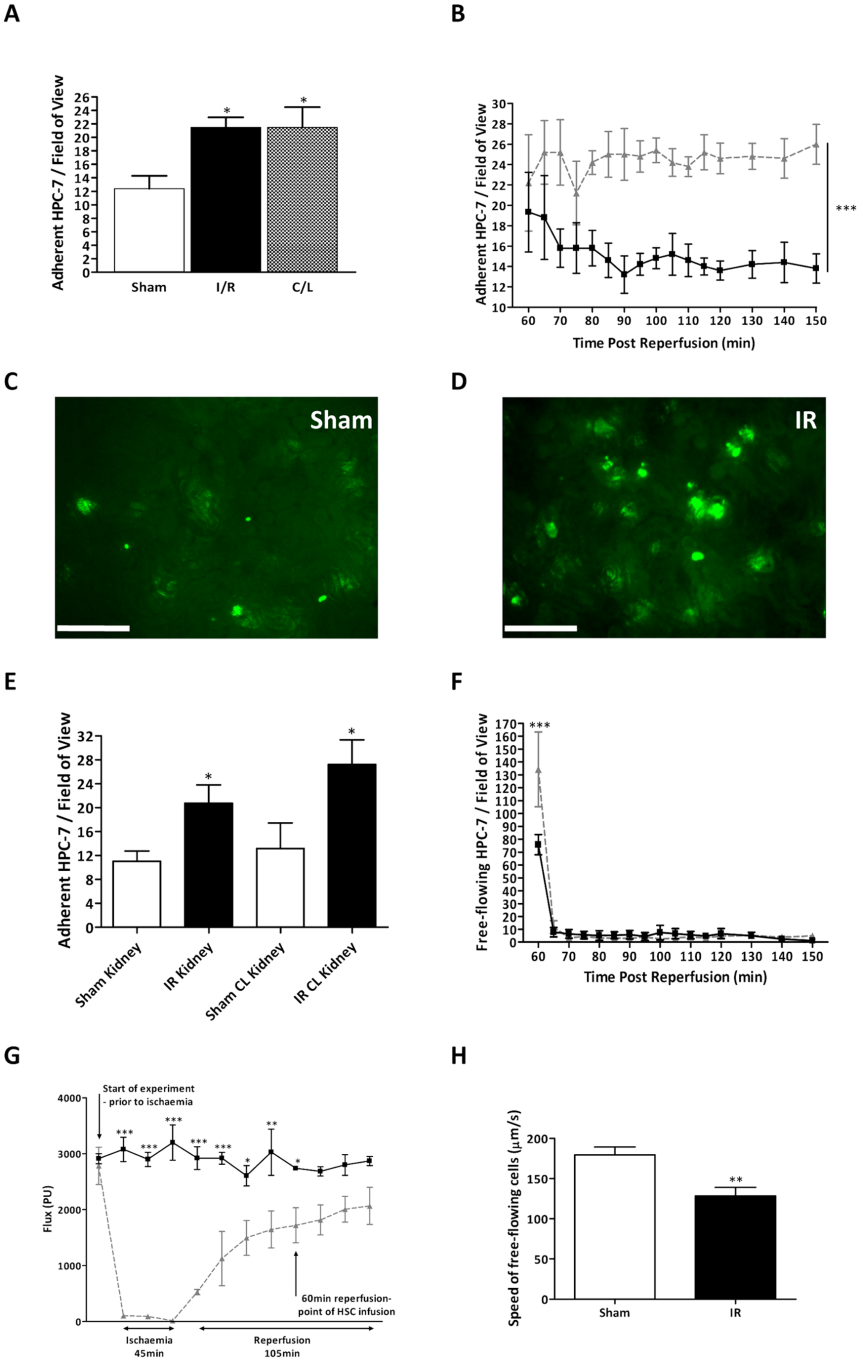
Adherent and free flowing HPC-7 s are increased in IR injured kidney. A significant increase in HPC-7 to frozen sections of IR injured and non-injured CL tissue was observed compared to controls (A). Similarly, adhesion *in vivo* within renal peritubular capillaries was also significantly increased in IR injured mice compared to controls (B). Representative images of CFSE-labeled HPC-7 s in sham (C) and IR-injured (D) renal microcirculation are shown; scale bars shown are 200 μm. Both in focus and out of focus cells are counted. These events were paralleled by those occurring in other randomly selected regions of the kidney (E). Free flowing HPC-7 numbers were increased in IR injured mice at the point of infusion (F). Blood flow was significantly reduced in IR injured mice at the time of HPC-7 infusion (G). HPC-7 velocity *in vivo* was significantly reduced in IR injured renal microcirculation compared to sham (H). For all line graphs: sham control  =  solid line; IR-injured  =  dashed line. Results are presented as mean ± SEM (n≥4). *p<0.05, **p<0.01, ***p<0.001.

Significantly (p<0.05) increased adhesion was observed *in vitro* on injured and contralateral (CL) renal sections taken from IR injured mice compared to sham tissue (**Figure 1A**). Adhesion was also significantly (p<0.001) increased within the peritubular microcirculation *in vivo* in injured animals compared to shams (AUC: Sham: 221.90±20.62; IR: 367.30±21.16; **Figures 1B–D**). Adhesive events observed in the single pre-selected area were paralleled by those occurring in other randomly selected regions of the kidney with significantly (p<0.05) increased adhesion in the injured and CL kidney of IR injured mice compared to sham (**Figure 1E**).

At the point of HPC-7 infusion (60 minutes post-reperfusion), the number of free-flowing cells were significantly (p<0.05) increased in injured animals compared to sham animals (**Figure 1F**). This effect was not seen at any other time point. Since this may result from increased renal blood flow (reactive hyperemia) following IR injury, laser speckle contrast microscopy was used to determine blood flow in sham and injured kidneys. At 60 minutes post-reperfusion, renal blood flow was significantly (p<0.05) decreased in injured mice compared to sham mice (Flux: sham kidney: 2739.57±21.97; IR kidney: 1719.20±312.97; **Figure 1G**). Furthermore, HPC-7 speed was also measured *in vivo* but was significantly (p<0.01) reduced in injured microcirculation compared to the microcirculation in sham animals (**Figure 1H**).

### HSC recruitment to IR injured kidney is dependent on CD44 and CD49d

**Figure 2:**
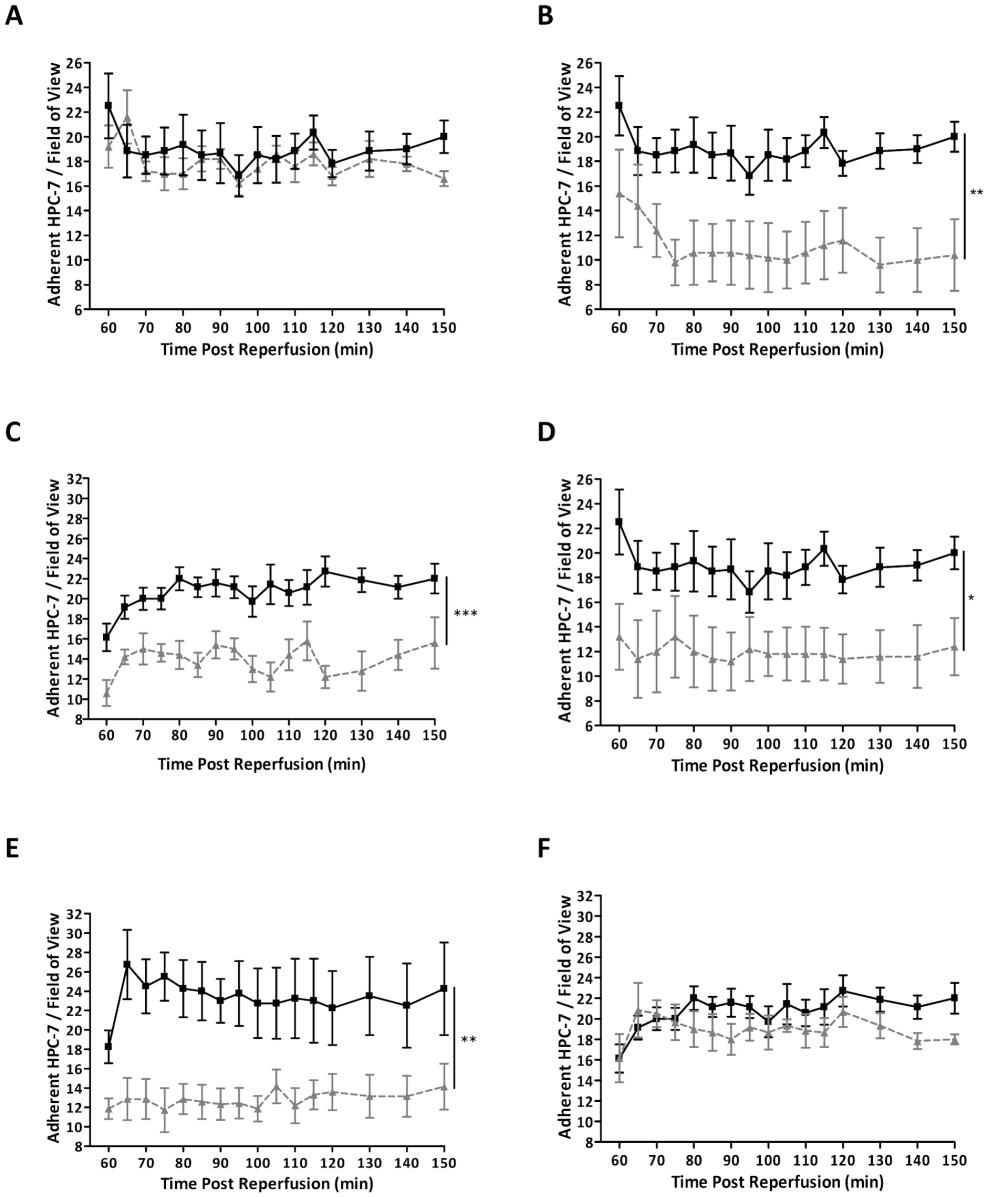
HPC-7 recruitment to IR injured kidney is dependant on CD44 and CD49d. 2×10^6^ HPC-7 were pre-treated with function-blocking monoclonal antibodies (80 µg/ml) against integrins CD18 and CD49d and the non-integrin CD44. Function blocking antibodies to endothelial VCAM-1 and CD44 and the enzyme hyaluronidase (to block HA) were administered *in vivo* at 1 minute post-reperfusion and 2×10^6^ naïve HPC-7 were infused at 60 minutes. No decrease in HPC-7 adhesion was observed with an anti-CD18 antibody compared to IgG control (A). Adhesion was significantly reduced by blocking CD49d (B) and also when its endothelial counter-ligand, VCAM-1, was blocked *in vivo* (C). Similarly, adhesion was significantly reduced by blocking CD44 on HPC-7 (D) and also when its endothelial counter-ligand, HA, was blocked *in vivo* (E). Blocking endothelial CD44 did not decrease HPC-7 adhesion (F). For all graphs: IgG controls  =  solid line; blocking treatments  =  dashed line. Results are presented as mean ± SEM (n≥4). *p<0.05, **p<0.01, ***p<0.001.

We previously demonstrated that HPC-7 express CD18, CD44 and CD49d on their surface [20]. Pre-treating HPC-7 s with a function blocking antibody against CD18 had no effect on their adhesion within injured kidney *in vivo* (**Figure 2A**). However, blocking CD49d significantly (p<0.01) decreased HPC-7 adhesion (AUC: IgG: 282.3±20.69; anti-CD49d: 164.9±36.68; **Figure 2B**). Furthermore, intra-arterial administration of an anti-VCAM1 antibody significantly (p<0.001) reduced HPC-7 adhesion when compared to intra-arterial administration of an IgG control (**AUC**: IgG: 312.6±15.79; anti-VCAM1: 209.9±16.06; **Figure 2C**). In addition, blocking CD44 significantly (p<0.05) decreased HPC-7 adhesion within injured kidney *in vivo* (AUC: Anti-CD44: 178.00±35.00; **Figure 2D**). The major endothelial counter-ligand for CD44 on HPC-7 appeared to be HA, as digestion of HA with hyaluronidase *in vivo* was associated with a significant (p<0.01) decrease in HPC-7 adhesion (AUC: PBS: 353.0±49.67; hyaluronidase: 190.9±24.53; **Figure 2E**). Blocking endothelial CD44 *in vivo* did not alter HPC-7 adhesion (**Figure 2F**).

### KC and SDF-1α mediate HSC recruitment to healthy and IR injured kidney

**Figure 3:**
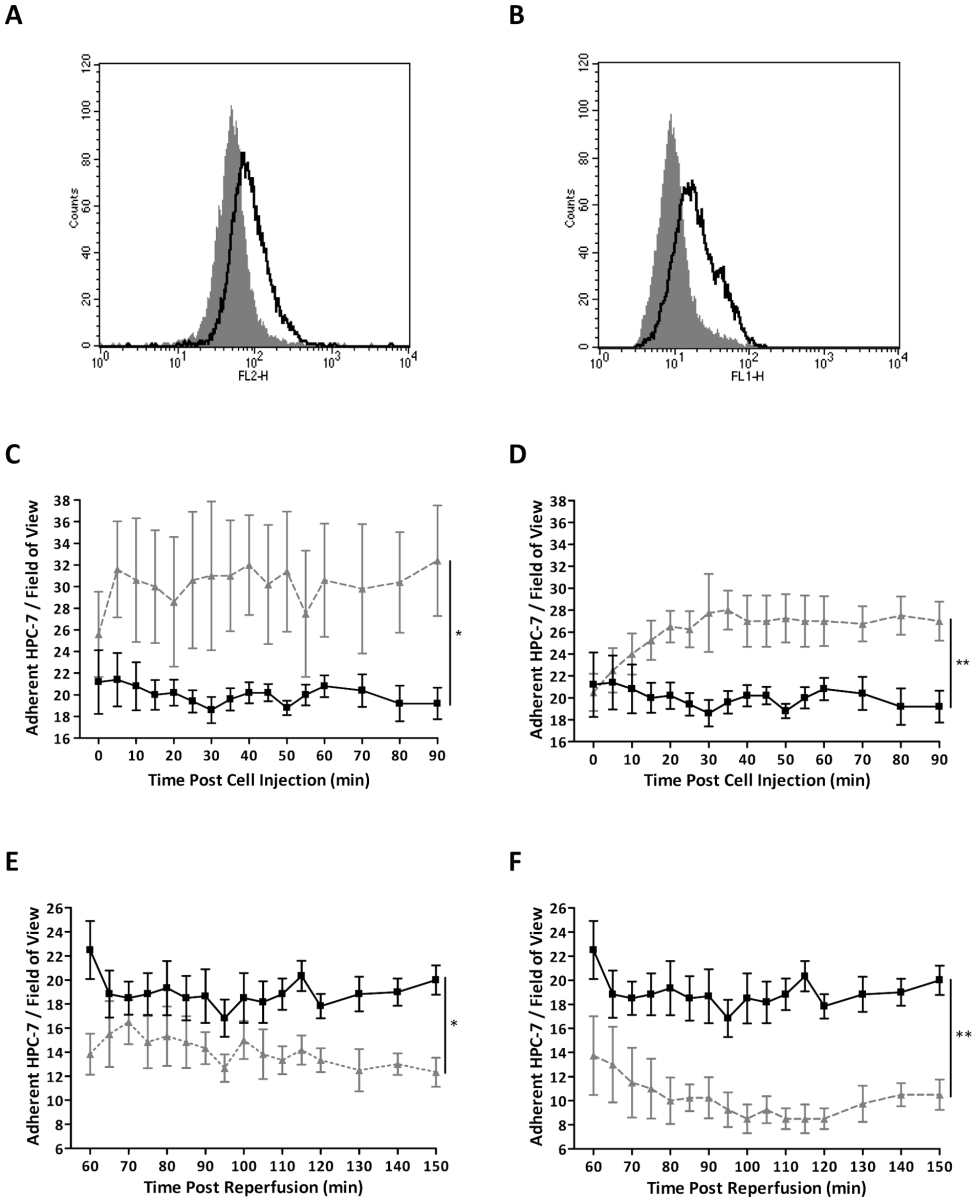
KC and SDF-1α mediate HPC-7 recruitment to the healthy and IR injured kidney. HPC-7 expressed the main KC and SDF-1α receptors, CXCR2 (A) and CXCR4 (B) respectively. Topically treating a healthy kidney with KC [200 ng/ml; C] or SDF-1α [200 ng/ml; D] for 4 hours led to significant HPC-7 recruitment compared to the PBS control. Blocking CXCR2 (E) and CXCR4 (F) on HPC-7 prior to administration also decreased HPC-7 adhesion *in vivo* within the IR injured kidney. For all figures: PBS controls  =  solid line; kidney pre-treatments/mAb treated HPC-7 =  dashed line. Results are presented as mean ± SEM (n≥4). *p<0.05, **p<0.01.

Flow cytometry demonstrated that CXCR2 (KC receptor) and CXCR4 (SDF-1α receptor) were expressed on HPC-7 (**Figures 3A–B**). Renal HPC-7 recruitment was significantly increased when healthy kidney was topically exposed to KC (p<0.05; **Figure 3C**) or SDF-1α (p<0.01; **Figure 3D**) when compared to PBS controls (AUC: PBS: 299.70±16.59; KC: 457.20±79.55; SDF-1α: 393.50±26.87). Increased adhesion was observed at 4 hours post-treatment with topical KC. However, adhesion following SDF-1α exposure was more gradual (**Figure 3D**). A role for KC and SDF-1α in mediating HPC-7 recruitment to injured kidney was also demonstrated as functionally blocking CXCR2 (p<0.05) or CXCR4 (p<0.01) on HPC-7 s significantly decreased adhesion within injured kidney *in vivo* compared to IgG control (**Figures 3E–F**). This effect was more pronounced when blocking CXCR4.

### HSC adhesion can be enhanced by pre-treatment with KC and SDF-1α

**Figure 4:**
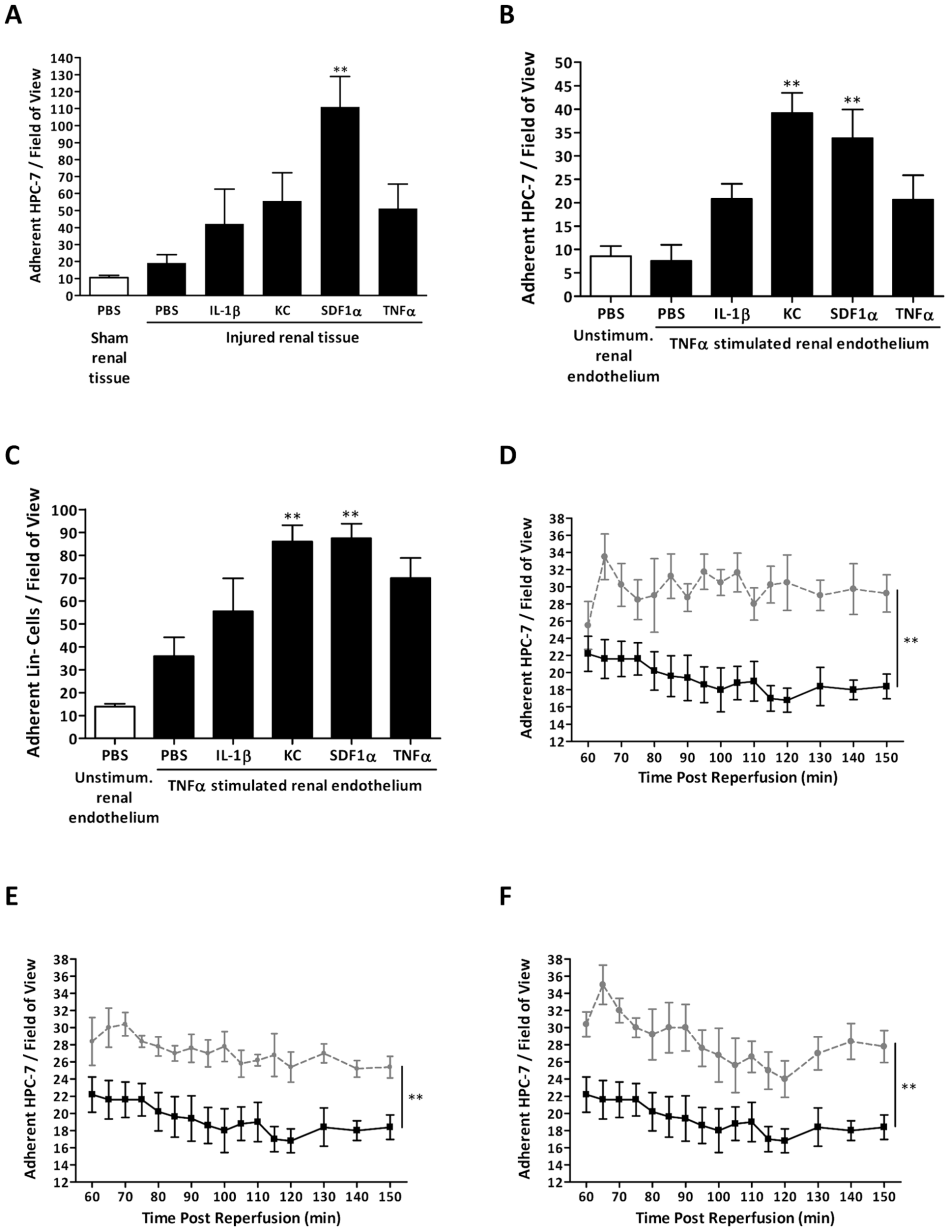
HPC-7 adhesion can be enhanced by pre-treating them with KC and SDF-1α. Only HPC-7 pre-treatment with SDF-1α (25 ng/ml; 5 minutes) significantly increased HPC-7 adhesion to IR injured frozen renal tissue when compared to PBS control (A). Pre-treatment with KC (25 ng/ml; 5 minutes) and SDF-1α (25 ng/ml; 5 minutes) significantly increased HPC-7 adhesion to TNFα (100 ng/ml; 4 hours) activated murine renal endothelium compared to the PBS control (B). Pre-treatment of primary murine lineage negative cells also yielded similar results. Both KC and SDF-1α significantly increased Lin^−^ cell adhesion to TNFα activated renal endothelium (C). Pre-treating with KC (D), SDF-1α (E) or KC+SDF-1α (F) significantly increased HPC-7 adhesion within IR injured kidney *in vivo*, although dual pre-treatment did not confer a greater effect. For line graphs: PBS pre-treated HPC-7+ IR kidney  =  solid line; KC and/or SDF-1α pre-treated HPC-7 s + IR injured kidney  =  dashed line. Results are presented as mean ± SEM (n≥4). **p<0.01.

Pre-treating HPC-7 with SDF-1α, but not IL-1β, KC or TNFα, for 5 minutes significantly (p<0.01) enhanced their adhesion to frozen sections of injured kidney when compared to PBS controls (**Figure 4A**). Pre-treating with KC (p<0.01) or SDF-1α (p<0.01) significantly enhanced adhesion to TNFα stimulated murine renal ECs (**Figure 4B**). Both KC (p<0.01) and SDF-1α (p<0.01) pre-treatment significantly enhanced adhesion of primary Lin^−^ cells to TNFα stimulated murine renal ECs (**Figure 4C**). Both KC and SDF-1α pre-treatment significantly (p<0.01) increased HPC-7 adhesion *in vivo* in injured kidney (AUC: PBS: 300.30±25.62; KC: 449.30±30.69; SDF-1α: 409.30±16.67; **Figures 4D–E**). However, no further increase in adhesion was observed when HPC-7 were pre-treated with both these chemokines together (**Figure 4F**).

### SDF-1α pre-treatment increases free flowing HSCs in IR injured kidney potentially through increased HSC deformability

**Figure 5:**
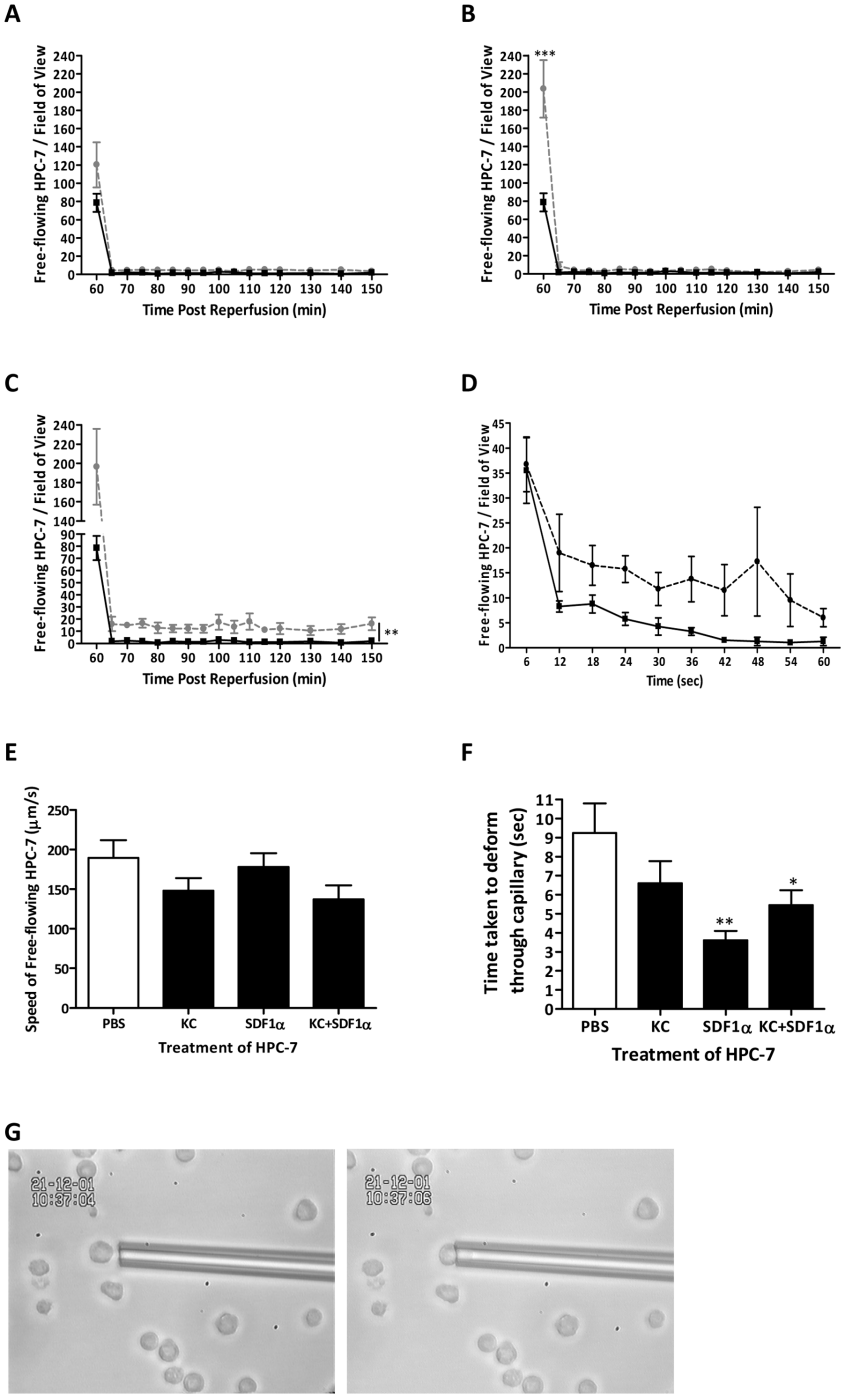
SDF-1α pre-treatment increases numbers of free flowing HPC-7 in IR injured kidney. Following KC pre-treatment, no difference in free flowing HPC-7 numbers was observed at anytime point in IR injured kidney (A). However, SDF-1α pre-treatment significantly increased free-flowing HPC-7 s at the point of administration only (B). Dual pre-treatment with both KC+SDF-1α not only increased free-flowing cells at the point of infusion but sustained this increase in HPC-7 trafficking around the renal microcirculation throughout the entire period of observation (C). The first one minute recording was broken down into 6 second intervals to illustrate each full circulatory pass. With SDF-1α pre-treatment, more HPC-7 continue to flow between 6–24 seconds suggesting less are lost to the circulation (D). HPC-7 velocity *in vivo* was not affected by any pre-treatment (E). HPC-7 became significantly more deformable with SDF-1α and KC+SDF-1α pre-treatment as demonstrated by reduced time taken to aspirate into a glass capillary (F). A photograph illustrating the method is shown and 50 cells were tested/group (G). For all line graphs; PBS treated HPC-7+ IR kidney  =  solid line; KC and/or SDF-1α treated HPC-7 s + IR injured kidney  =  dashed line. Results are presented as mean ± SEM (n≥4). *p<0.05, **p<0.01.

Pre-treating HSCs with KC did not significantly increase numbers of free-flowing cells trafficking through the injured kidney compared to PBS pre-treated cells (**Figure 5A**). Interestingly, significantly (p<0.01) increased free-flowing HPC-7 were observed immediately upon infusion within injured renal microcirculation with SDF-1α pre-treatment (**Figure 5B**). This effect was not observed at any other time point. Dual pre-treatment of HPC-7 with both KC and SDF-1α led to significantly (p<0.01) increased numbers of freely flowing cells observed at all time points (AUC: PBS: 54.80±12.50; KC+SDF-1α: 282.30±60.53; **Figure 5C**).

Free flowing cells were defined as the total number of cells observed flowing through the field of view in a 1 minute time-frame of continuous observation. The blood circulation time for a mouse is approximately 4–6 seconds, meaning blood circulates approximately 10 times during a 1 minute observation period. To determine whether SDF-1α pre-treated HPC-7 only passed once through the kidney or if they were re-circulated, numbers of freely flowing cells at 6 second intervals were determined for the first observation minute. Increased freely circulating HPC-7 were observed at each 6 second time point for the first minute following SDF-1α pre-treatment, suggesting more were continually re-circulated, with less being lost to extra-renal sites (**Figure 5D**).

No significant change in HPC-7 size was observed as a result of chemokine pre-treatment, assessed using a coulter counter (data not shown). The velocity of pre-treated HPC-7 in microvessels was also investigated using the offline Slidebook analysis program, but no significant changes were noted (**Figure 5E**). SDF-1α (p<0.01) and KC+SDF-1α (p<0.05) treated cells were significantly resistant to flow, as determined by a reduction in the time taken for cells to be fully aspirated into a glass micropipette (diameter comparable to blood capillaries). SDF-1α alone caused a 61% reduction in entry time (**Figure 5F–G**).

### KC and SDF-1α pre-treatments cause the lateral movement of HSC surface receptors, which may explain the increase in adhesion

**Figure 6:**
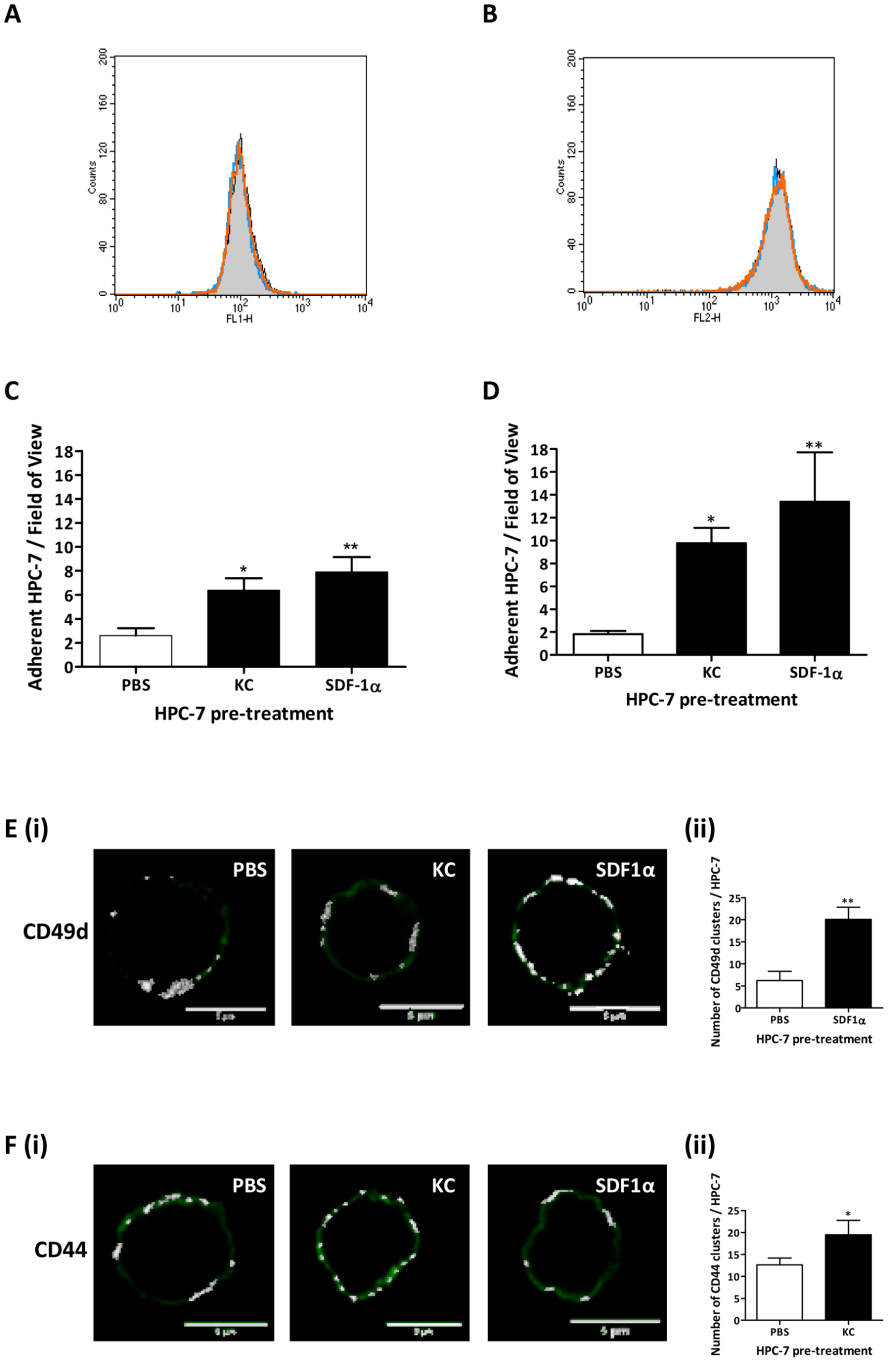
KC and SDF-1α pre-treatment increases adhesion of HPC-7 surface adhesion molecules for their endothelial counter-ligands. Pre-treatment with KC or SDF-1α did not increase surface expression of CD49d (A) or CD44 (B) on HPC-7 s. PBS-treated HPC-7 =  black line, grey fill; KC-treated HPC-7 =  blue line; SDF1α-treated HPC-7 =  orange line. Both KC and SDF-1α pre-treatment significantly increased HPC-7 adhesion to immobilised VCAM-1 (C) and HA (D) when compared to PBS pre-treated control cells. SDF-1α pre-treatment only increased the number of clusters of CD49d on the HPC-7 surface (Ei–ii). However, KC pre-treatment only increased the number of clusters of CD44 on the HPC-7 surface (Fi–ii). Results are presented as mean ± SEM (n≥3). *p<0.05, **p<0.01, ***p<0.001.

Flow cytometry demonstrated treating HPC-7 with KC or SDF-1α did not alter cell surface expression of either CD49d or CD44 (**Figures 6A–B**). Nevertheless, KC and SDF-1α pre-treatment significantly increased HPC-7 adhesion to immobilised VCAM-1 and HA respectively when compared to PBS pre-treated controls (**Figures 6C–D**). Furthermore, SDF-1α significantly (p<0.01) increased the number of CD49d clusters (**Figures 6E** **i–ii**). In addition, KC significantly (p<0.05) increased the number of CD44 clusters (**Figures 6F** **i–ii**) on the HPC-7 surface.

## Discussion

Although HSCs are beneficial for a variety of renal disorders, their efficacy is likely to depend on successful local recruitment [7], [8], [9], [33]. Improving the efficacy of regeneration may therefore depend on identifying adhesive mechanisms that underpin SC trafficking. However, no studies have previously described the mechanisms governing renal HSC recruitment. We developed the intravital imaging methodology to obtain valuable insights into the kinetics of stem cell homing within the mouse renal microcirculation immediately following their infusion. This study provides the first direct *in vivo* evidence that HSC recruitment to acutely injured kidney is a regulated event and that, more importantly, recruitment achieved by injury alone is not maximal.

HPC-7 adhesion within injured kidney is dependent upon CD44 and CD49d and we further demonstrated that VCAM-1 and HA, known to be up-regulated in IR injured kidney [34], [35], [36] were the endothelial counter-ligands for CD49d and CD44 respectively. In addition to HA, CD44 has also been shown to interact homotypically with CD44 expressed on endothelial cells [37]. However, while CD44 is up-regulated on renal capillary ECs after IR injury [38], we found that CD44 homotypic interactions did not govern HSC adhesion in our renal IR injury model. We have previously shown a critical role for CD49d in mediating HSC recruitment to the liver [20], indicating that this adhesion molecule may have a dominant role to play in HSC homing to injured sites. However, HSC recruitment to injured gut has been shown to be dependent on the β_2_ integrin subunit CD18 (Kavanagh et al., under review), suggesting there is also a degree of site specificity with regards the adhesive mechanisms involved. Understanding this site-specificity is particularly important when developing strategies to target SC adhesion within specific organs. Interestingly, studies suggest the CD44/HA pathway also governs mesenchymal stem cell (MSC) recruitment in the glycerol-induced model of acute renal injury [39]. This study utilised immunohistochemistry and electron microscopy to demonstrate that only CD44^+/+^ and not CD44^−/−^ MSCs could be located within the renal cortex. More importantly, it demonstrated that MSCs lacking CD44 could not be recruited to the kidney, and that this resulted in a loss of therapeutic renal benefit. This clearly demonstrates that the active local recruitment of SCs using surface adhesion molecules, such as CD44, is an essential pre-requisite for their beneficial effect.

Since HSC adhesion molecules play an important part in HSC-endothelial interactions, we hypothesised that modulating their expression and/or capacity to bind their endothelial counter-ligands might be an important approach to improve HSC renal homing. Chemokines such as SDF-1α and KC can mediate adhesion of circulating leukocytes, so we investigated whether both of these could also influence HPC-7 adhesion. Using a novel preparation that immerses the exteriorsed kidney in chemokine, we demonstrated that SDF-1α, and interestingly KC, could promote HSC adhesion within a healthy kidney. Furthermore, both chemokines played a significant role in modulating adhesion in the injured kidney, as blocking CXCR4 and CXCR2 inhibited their recruitment. SDF-1α has also been shown to play an important role in mediating the homing of CXCR4^+^ BM-derived cells in IR injured kidney [40]. Interestingly, Stroo *et al.* showed that manipulating the SDF-1α/CXCR4 axis, either by increasing local SDF-1α concentrations in the injured kidney or by blocking CXCR4 on HSCs, did not affect their migration [41]. However, in these studies, they injected recombinant SDF-1α into just one focal point in the kidney, which may explain this discrepancy. Although there is a strong association described between SDF-1α and SC homing [42], [43], it was interesting to observe that HSC adhesion to the KC-exposed healthy kidney was more rapid than SDF-1α treatment. This is the first time a novel role for this classical neutrophil chemoatttractant has been directly demonstrated in mediating SC recruitment in a tissue bed.

Having demonstrated critical roles for SDF-1α and KC, we generated novel data showing that pre-treatment with either can significantly increase HSC homing. We also demonstrated that the HPC-7 cell line adhesive behaviour to chemokine pre-treatment is similar to primary Lin^−^ cells. This is the first study to show that HSC adhesion within the injured kidney can be enhanced above that seen with injury alone. Others have shown, for example, that MSC homing to bone could be increased using cells genetically engineered to express higher surface levels of CD49d [44]. In our study, enhanced adhesion was most likely mediated by enhanced CD49d and CD44 binding to VCAM-1 and HA respectively. Furthermore, we provide evidence that both chemokines induced surface clustering of CD49d and CD44. Grabovsky and colleagues demonstrated that the close proximity between chemokine receptors and surface integrins facilitates the rapid conversion of a signaling event into integrin clustering in leukocytes [45]. Although conformational changes in adhesion molecules from an inactive to active state are important for mediating cell adhesion, the dynamic reorganisation of adhesion molecules into clusters is also a major mechanism that regulates their binding capacity, acting to strengthen cell-cell adhesion [46].

Using the micropipette aspiration technique, we have provided the first evidence that chemokines could also alter HSC deformability. It is possible that this decreased resistance to deformation prevented circulating cells from becoming entrapped within non-renal sites and maintained their presence within peripheral blood. Non-specific entrapment is a major obstacle for systemic SC delivery for regenerative purposes both experimentally and clinically [47] and significantly reduces the pool of circulating transplanted HSCs available for recruitment. However, at the time of infusion, SDF-1α pre-treated HSCs were observed to repeatedly circulate unlike PBS pre-treated cells, which were rapidly lost from the peripheral circulation. KC and SDF-1α dual pre-treated cells continuously trafficked through the kidney throughout the duration of the experiment, with approximately 10–15 cells observed at each time point. This phenomenon may increase the chance of trafficking cells becoming adherent within injured renal microvessels and also contribute to the enhanced adhesion observed.

It was interesting to observe an increase in HSC binding within the non-injured contralateral kidney. IR injury causes the kidney to release a number of soluble inflammatory factors (TNFα, TNFβ, and IL-6 amongst others) into the systemic circulation which could lead to up-regulation of adhesion molecules on endothelium of remote sites [48]. Lin and colleagues in 2003 noted donor-derived HSCs in the non-injured contralateral kidney, suggesting systemic factors can modulate HSC adhesion [7]. Interestingly, these results do question the use of the non-clamped contralateral kidney as an internal control in many others types of studies assessing renal injury [49], [50].

In conclusion, despite huge advances in the field of cellular therapy, a major impediment remains their poor retention within target tissues on systemic delivery. We provide a simple, quick and effective method for enhancing HSC adhesion into injured kidney using SDF-1α or KC. These chemokines most likely mediate increased recruitment either through the modulation of CD49d and/or CD44, or by reducing HSCs clearance from the circulation. Previous studies have enhanced SC recruitment by introducing genes encoding for SDF-1α within cardiac tissue [51]. However, the clinical applicability of such techniques is debatable and may be associated with aggravated tissue injury due to side effects such as SDF-1α-dependent lymphocyte recruitment [52]. The current study benefits from identifying a strategy that increases recruitment without genetically manipulating the HSCs or the host tissue and thus has the potential to be used clinically. Our data may therefore help in the design of future cellular therapies using HSCs for renal repair. It is anticipated that enhancing HSC recruitment to injured kidney may expedite the recovery process and encourage greater therapeutic success clinically. Further detailed studies will be required to determine this and are currently underway.

## Materials and Methods

### Animals

Male C57BL/6 (8–12 week old; Harlan, UK) were used for procedures in accordance with the Animals (Scientific Procedures) Act of 1986 (Project Licence: 40/3336) and was approved by the local Birmingham Ethical Review Sub-Committee (BERSC) prior to Home Office approval. All procedures were conducted under terminal anaesthesia with animals being sacrificed by cervical dislocation, as approved by the Home Office. Anaesthetised animals (ketamine/xylazine; ip.) underwent tracheostomy to assist breathing and carotid artery cannulation to allow administration of labelled cells and any additional anaesthetic. Following a midline laparotomy, renal ischaemia was induced by clamping the left renal pedicle for 45 minutes, with removal of the clamp initiating reperfusion. Control animals underwent sham surgery.

### Cells

Intravital studies monitoring HSC trafficking *in vivo* to sites of injury have been limited due to difficulties in isolating sufficient numbers for detection following systemic infusion. We have found that approximately 5,000 primary murine HSCs, defined as being c-Kit^+^, Sca-1^+^ and lineage negative (Lin^−^; KSL cells), can be obtained from one adult mouse. This is too low for intravital or even *in vitro* adhesion assays – pooling cells from mice would require an unacceptable number of donor mice to be culled for a individual intravital experiments. Therefore, an embryonic murine HSC line, HPC-7, were used and were kindly given as a gift from Professor L. Carlsson, Umea University, Sweden. These cells (HPC-7) have been used extensively in published studies [20], [29], [53], [54], [55], [56]. HPC-7 are an immortalised SC line generated by transfection with the LIM-homeobox gene, Lhx2, into murine embryonic SCs [56]. This cell line displays many of the critical characteristics of primary HSCs, including expression of c-kit and Sca-1 on their surface and also being lineage negative. Crucially, HPC-7 are able to fully reconstitute haematopoiesis when injected into a lethally irradiated host [55]. Furthermore, HPC-7 express surface adhesion molecules known to be expressed on primary HSCs and we have previously used them to model hepatic and intestinal recruitment intravitally [20], [29]. Cells were maintained in Stem Pro 34 SFM supplemented with the manufacturer's media (Life Technologies, UK), L-glutamine, penicillin and streptomycin (PAA, Somerset, UK). HPC-7 s were fluorescently labelled with 5(/6)-carboxy-fluorescein diacetate, succinimidyl ester prior to use (CFSE; Life Technologies, UK).

To confirm that the HPC-7 line responded to our chemokine pre-treatment strategies in a similar way to primary cells, we isolated murine Lin^−^ BM-derived cells for use in adhesion assays. This particular subset of cells were selected as it is possible to yield sufficient cell numbers from three donor mice to perform static adhesion assays. In addition, Lin^−^ cells have been shown to be therapeutic in chronic kidney disease [57]. Briefly, whole BM cells were obtained from the femurs and tibias of C57Bl/6 mice and depleted for lineage positive cells using biotinylated lineage cocktail antibodies (CD5, CD45R (B220), CD11b, Gr-1 (Ly-6G/C), 7–4 and Ter-119) and magnetic activated cell sorting in accordance with the manufacturer's instructions (MACS; Mitenyi Biotech).

### Antibodies

Flow cytometry studies were conducted using fluorescein isothiocyanate (FITC)-conjugated isotype control rat IgG2b, phycoerythrin (PE)-conjugated isotype control IgG2b, FITC-anti-CD18 (GAME-46; Santa Cruz), PE-anti-CD44 (IM7), FITC-anti-CD49d (MFR4.B), FITC-CD184 (CXCR4; 2B11; eBioscience, San Diego, California, USA) and Phycoerythrin (PE)-conjugated rat monoclonal anti-mouse CXCR2 (242216; R&D Systems, UK). Low endotoxin, axide-free anti-CD18 (GAME-46), anti-CD44 (IM7), anti-CD49d (R1-2), anti-CD106 (429/MVCAM.A), and isotype control rat IgG2a antibodies were used for function blocking studies (Cambridge Biosciences, UK; anti-CD18 from BD Pharmingen, UK; hyaluronidase from Sigma, UK). Type VI-S hyaluronidase was used to degrade endogenous HA (Sigma-Aldrich, UK). HPC-7 were treated with 80 μg/ml of blocking antibody for 30 min in PBS containing 0.1% bovine serum albumin (Sigma, UK) for HSC blocking studies. Anti-CXCR2 (242216; R&D Systems, UK) and anti-CXCR4 (2B11; R&D Systems, UK) function blocking monoclonal antibodies were used to block chemokine receptor activity on HPC-7 at concentrations of 50 μg/ml and 40 μg/ml respectively. For some studies, animals received a bolus injection of 100 μg of anti-CD44 mAb, 70 μg of anti-VCAM-1 mAB or 10 mg hyaluronidase. Control animals received an intra-arterial bolus of the corresponding dose of rat IgG2a (Cambridge Biosciences, UK) or PBS (Sigma, UK).

### Cytokines

Recombinant murine interleukin-1β (IL-1β), KC, SDF-1α and tumour necrosis factor-α (TNFα) were used to pre-treat HPC-7, Lin^−^ cells or exposed kidney (Peprotech, UK). Cytokines were made at a stock concentration of 10 µg/ml in PBS containing 0.1% bovine serum albumin (Sigma-Aldrich, Poole, UK) and were used experimentally at 25 ng/ml for HPC-7/Lin^−^ cell pre-treatment or 200 ng/ml for topical applications.

### Flow cytomtery

HPC-7 were analysed using flow cytometry to identify changes in cell surface expression of CD18, CD44, CD49d, CXCR2 and CXCR4. 1×10^6^ HPC-7(in 100 µl) were pre-treated for 5 minutes with PBS or 25 ng/ml KC and/or SDF-1α. Subsequently, cells were fixed with 5% formalin (Sigma-Aldrich, UK) at room temperature for 20 minutes. HPC-7 were blocked with 1 µg/ml low-endotoxin azide-free (LEAF) CD16/32 function blocking antibody (93; Biolegend, UK) to reduce subsequent non-specific antibody binding. Cells were then incubated with fluorochrome-conjugated primary antibody at 1:50 for 30 minutes at 4°C. Fluorescence cells were subsequently interrogated using flow cytometry on a BD FACSCalibur cytometer (Becton Dickinson, USA). Data was analysed with CellQuest (Becton Dickinson, USA).

### Frozen tissue section adhesion assay

Stamper-Woodruff assays were performed as previously described [58]. Briefly, the experimental and contralateral kidneys were isolated and snap frozen from sham and IR treated animals and sectioned using a cryostat to a thickness of 10 µm. 1×10^5^ CFSE-labelled HPC-7 were added to each section for 15 minutes and then washed with PBS to remove unbound cells. Sections were fixed in acetone, mounted and analysed microscopically. Adherent cells from 5 randomly selected fields of view were counted and the mean obtained.

### Endothelial cell static adhesion assay

Isolation of primary murine endothelial cells (ECs) is typically very difficult and requires elaborate and time consuming purification techniques [59]. Therefore, immortalized murine renal ECs (gift from Dr. J. Steven Alexander, LSU-HSC, USA) were cultured to confluence in gelatin-coated wells in a 24 well plate (BD Biosciences) [60]. At confluence, monolayers were treated for 4 hours with 100 ng/ml TNF-α (Peprotech, UK). Following washing with 0.1% bovine serum albumin in PBS, 1×10^5^ HPC-7 cells (in 500 µl) were added for 20 minutes. Some HPC-7 were pre-treated with IL-1β, KC, SDF-1α or TNFα (25 ng/ml in complete Stem Pro 34 SFM) for 5 minutes. Wells were washed with PBS and fixed with 2% glutaraldehyde (Sigma, UK) in PBS for 15 minutes at 37°C. Adherent cells from 5 random fields were counted using fluorescent microscopy (Olympus IX81; Olympus, UK) and the mean obtained.

### Immobilised counter-ligand adhesion assay

96-well plates (Nunc, Rochester, USA) were coated with 10 µg/ml of recombinant murine (rm) ICAM-1 Fc chimera, rmVCAM-1 Fc chimera (R&D Systems, UK) or 0.5 mg/ml Hyaluronan (rmHA; Sigma, UK) for 1 hour. Wells were then washed, blocked using 10 mg/ml heat-denatured BSA and incubated with pre-treated HPC-7 at 37°C for 20 minutes. HPC-7 were pre-treated for 5 minutes at 37°C with 25 ng/ml KC or SDF-1α (made up in complete Stem Pro 34 SFM; Peprotech, UK). Prior to pre-treatment HPC-7 were blocked with 1 µg/ml low-endotoxin azide-free (LEAF) CD16/32 function blocking antibody (Biolegend, UK). Adherent HPC-7 were fixed by incubation with 2% glutaradehyde (Sigma-Aldrich) at 37°C for 15 minutes. Wells were washed and 100 µl PBS was added for imaging adherent cells on an inverted microscope.

### Confocal microscopy for monitoring receptor clustering

HPC-7 were treated for 5 minutes with 25 ng/ml KC and/or SDF-1α at 37°C and then fixed for 15 minutes at room temperature. Cells were washed and subsequently incubated with primary antibody (anti-CD49d and anti-CD44) for 1 hour on ice. Cells were washed again and incubated with a secondary antibody (Alexa Fluor® 488 goat anti-rat IgG H+L; Life Technologies, UK) for 30 minutes on ice. Cells were added to air-dried washed lysine coverslips for 1 hour. These were mounted onto coverslides ready for imaging using confocal microscopy (Leica TCS-SP2) and 90 cells were imaged per treatment group.

### Intravital microscopy

This imaging modality is frequently used to visualise the microcirculation of solid organs. However, using intravital microscopy for imaging the renal microcirculation in mice is challenging and as a result, there are limited studies of this nature. This is primarily due to the difficulties associated with exteriorising the mouse kidney, minimizing the effects of respiratory movements and having the correct optics and cameras to get high resolution, dynamic, real-time images. We developed the methodology to investigate the kinetics and molecular adhesive events involved in mediating HSC recruitment in the mouse kidney. Following reperfusion or sham surgery, the left kidney was exteriorised via a flank laparotomy. One field of view was randomly selected prior to HPC-7 administration and continually monitored intravitally in order to establish HPC-7 adhesion dynamics within peritubular capillaries (×10; Olympus IX81). For some studies, the exteriorised healthy non-injured kidney was topically treated for 4 hours with 200 ng/ml of KC, SDF-1α or PBS using a specially designed container that allowed the whole kidney to be bathed with solution. 2×10^6^ CFSE-labelled HPC-7 were administered systemically in a single 100 µl bolus at 60 min post-reperfusion or at 4 hours post-chemokine exposure. For some studies, cells were pre-treated with blocking antibodies or chemokines. 1 minute recordings were made every 5 minutes for a total of 90 minutes. Cells were quantitated as either free-flowing or adherent (static for ≥30 s). At the end of the experiment, images from five additional randomly selected fields of view were taken to ensure that the events taking place in the pre-selected view were representative. Digital images were analysed off-line (Slidebook; Intelligent Imaging Innovations, USA). The mean velocities (μm/s) of cells trafficking through the renal microvasculature were also calculated using Slidebook imaging software.

### Blood flow measurements

We combined intravital microscopy with laser speckle microscopy in order to correlate the HSC adhesive events with renal blood flow. The Moor FLPI laser speckle camera (Moor Instruments, UK) was used to determine renal surface blood flow within exposed IR injured and sham kidneys as described previously [61]. Recordings were made every 15 minutes throughout the ischemic and reperfusion phases and an arbitrary unit of flux was obtained.

### Micropipette assay

The deformability of HPC-7 following chemokine pre-treatment was assessed using a micropipette assay as previously described [62]. Briefly, PBS or cytokine treated HPC-7 were placed in a chamber made up of two coverslips separated by a U-shaped gasket and placed on a microscope stage held at 37°C. A micropipette with an internal diameter of approximately 5 μm was introduced into the chamber from the open side, and a fixed aspiration pressure of 1000Pa (10cmH_2_O) was applied by lowering a water reservoir connected to the pipette. Video recordings were made of HPC-7 aspirated into the pipette and the time taken to enter the pipette was measured offline. Typically 50 cells were tested in each sample.

### Statistics

Unpaired Student t-tests were used to make comparisons between two experimental groups for static adhesion assays, FACS data, changes in velocity and deformability. For more than two experimental groups, a one-way ANOVA followed by Dunnett's post-tests were carried out. For *in vivo* experiments, area-under-curve (AUC) calculations were performed to identify any significant differences between treatments. Analysis of free-flowing data at the point of cell infusion was calculated using an unpaired t-test. Blood flow data was analysed using a two-way ANOVA with Bonferroni post-tests. Values were considered significant when p<0.05.
